# Supplementary material for: Structure-Dependent Mechanism of Organophosphate Release from Albumin and Butyrylcholinesterase Adducts When Exposed to Fluoride Ion: A Comprehensive In Silico Study
Source: Int J Mol Sci. 2023 Oct 1;24(19):14819. doi: 10.3390/ijms241914819 (PMC10573431; doi:10.3390/ijms241914819)
Supplement: Supplementary file 1 [file ijms-24-14819-s001.zip › ijms-2639490-supplementary.pdf]

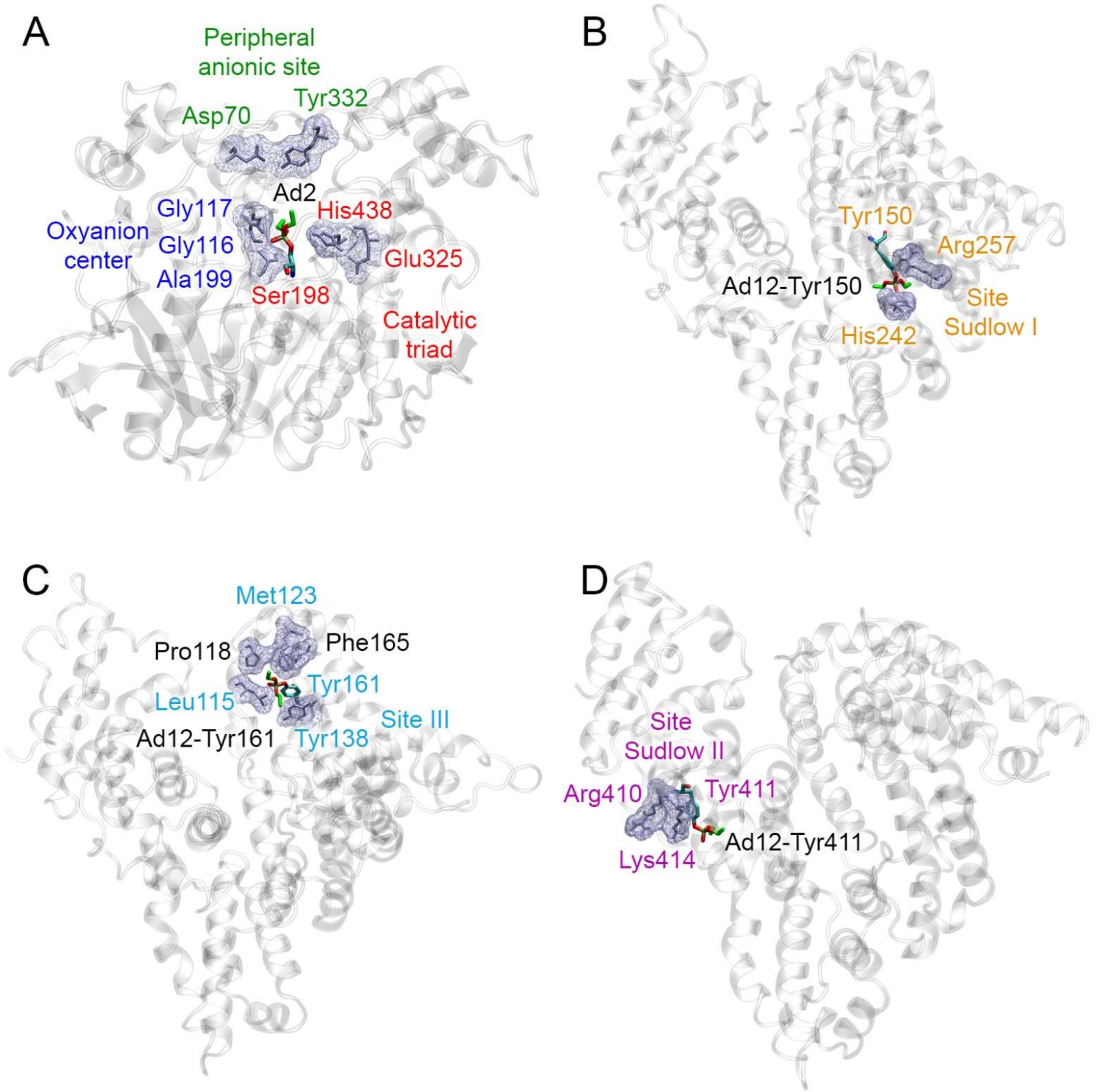

Figure S1. The topology of modified human butyrylcholinesterase (BChE) and human serum albumin (HSA).

A, structure of modified Ser 198 of BChE on the example of adduct Ad2. Amino acids of the catalytic triad, oxyanion hole and peripheral anionic site are highlighted in red, blue and green, respectively.

B, structure of modified Tyr150 of HSA on the example of adduct Ad12-Tyr150. Amino acids of site Sudlow I are highlighted in orange.

C, structure of modified Tyr161 of HSA on the example of adduct Ad12-Tyr161. Amino acids of site III are highlighted in light blue.

D, structure of modified Tyr411 of HSA on the example of adduct Ad12-Tyr411. Amino acids of site Sudlow II are highlighted in purple.
